# A review of the relationship between eating behavior, obesity and functional brain network organization

**DOI:** 10.1093/scan/nsz085

**Published:** 2019-11-04

**Authors:** Shannon D Donofry, Chelsea M Stillman, Kirk I Erickson

**Affiliations:** Department of Psychiatry, University of Pittsburgh School of Medicine, Pittsburgh, 15260, PA, USA; Department of Psychology, University of Pittsburgh, Pittsburgh, 15213, PA, USA; Department of Psychology, University of Pittsburgh, Pittsburgh, 15213, PA, USA; Department of Psychology, University of Pittsburgh, Pittsburgh, 15213, PA, USA; The Center for the Neural Basis of Cognition, Pittsburgh, 15213, PA, USA; Discipline of Exercise Science, College of Science, Health, Engineering and Education, Murdoch University, Western Australia, 6150, Australia

**Keywords:** obesity, disordered eating, functional brain networks, functional connectivity, resting state

## Abstract

Obesity is a major public health issue affecting nearly 40% of American adults and is associated with increased mortality and elevated risk for a number of physical and psychological illnesses. Obesity is associated with impairments in executive functions such as decision making and inhibitory control, as well as in reward valuation, which is thought to contribute to difficulty sustaining healthy lifestyle behaviors, including adhering to a healthy diet. Growing evidence indicates that these impairments are accompanied by disruptions in functional brain networks, particularly those that support self-regulation, reward valuation, self-directed thinking and homeostatic control. Weight-related differences in task-evoked and resting-state connectivity have most frequently been noted in the executive control network (ECN), salience network (SN) and default mode network (DMN), with obesity generally being associated with weakened connectivity in the ECN and enhanced connectivity in the SN and DMN. Similar disruptions have been observed in the much smaller literature examining the relationship between diet and disordered eating behaviors on functional network organization. The purpose of this narrative review was to summarize what is currently known about how obesity and eating behavior relate to functional brain networks, describe common patterns and provide recommendations for future research based on the identified gaps in knowledge.

## Introduction

Within the last several decades, the prevalence of obesity has risen dramatically, with over one-third of American adults meeting clinical criteria for the condition [body mass index (BMI) ≥ 30; Ogden *et al.*, 2014]. Numerous studies have demonstrated an adverse effect of having overweight or obesity on health and psychosocial functioning (Kopelman, 2000; Wadden and Stunkard, 2002; Finkelstein *et al.*, 2005; Cawley and Meyerhoefer, 2012), making the rise in rates of obesity a significant threat to public health. Obesity is associated with higher mortality and increased rates of cardiovascular disease, diabetes, hypertension, metabolic syndrome, depression (Stunkard *et al.*, 2003), anxiety (Gariepy *et al.,* 2010) as well as neurological illnesses such as Alzheimer’s disease (Kivipelto *et al.,* 2005). Further, individuals with overweight and obesity are more likely to experience discrimination in employment, education and health care settings (Cawley and Meyerhoefer, 2012). Obesity is one of the most costly conditions to treat, with obesity-related illness comprising up to 21% of yearly healthcare expenditures in the USA (Cawley and Meyerhoefer, 2012). These findings highlight the fundamental importance of identifying mechanisms underlying obesity to facilitate the development of more effective prevention and treatment interventions.

Obesity is associated with deficits in executive functioning, suggesting that functional differences in the brain regions that support these functions may contribute to the etiology of obesity. Executive function is defined as the ability to select appropriate actions based on ongoing evaluation of environmental demands, current goals, current emotional state, past experiences and social norms and encompasses elements of decision making and behavioral self-regulation (e.g. control of reward-driven impulses; Baumeister and Heatherton, 1996; Heatherton and Wagner, 2011). There is evidence that components of executive functioning, such as self-regulation, as well as the exertion of control over reward-driven impulses may be impaired in obesity (Schag *et al.*, 2013). For instance, individuals with obesity exhibit difficulty inhibiting automatic responses on inhibitory control tasks, tending to engage in habitual or overlearned behaviors (Batterink *et al.,* 2010; Davis *et al.*, 2010; Gunstad *et al.,* 2007). Individuals with obesity also show a preference for smaller, immediate rewards over larger, delayed rewards relative to normal weight individuals (Weller *et al.,* 2008). Importantly, steeper discounting of delayed future rewards has been associated with increased purchasing (Nederkoorn *et al.*, 2009) and consumption (Nederkoorn *et al.,* 2009; Appelhans *et al.,* 2011) of highly palatable, calorie-dense foods, as well as binge eating disorder (BED) (Davis *et al.,* 2010). These findings indicate that deficits in self-regulation and reward processing may predispose some individuals to obesity by influencing decisions regarding diet and exercise.

Poor self-regulation has also been shown to predict the probability of treatment failure during a weight loss intervention, with more impulsive individuals losing less weight than those with a greater capacity for self-regulation (Nederkoorn *et al.*, 2007). Indeed, greater capacity for self-regulation has been associated with more frequent consumption of healthy low-calorie foods and regular engagement in physical activity, both in healthy weight adults and adults with obesity (Wills *et al.*, 2007; Gerrits *et al.*, 2010; Crescioni *et al.*, 2011). Conversely, impaired self-regulation is associated with consumption of high-calorie foods and sedentary lifestyle, even during concerted weight loss attempts (Crescioni *et al.,* 2011). Moreover, one study demonstrated that specifically targeting deficits in self-regulation in a weight loss intervention promoted long-term maintenance of weight loss (Wing *et al.,* 2006). These data highlight the central role of self-regulation and reward processing in the etiology of obesity. These intervention studies also provide evidence that deficits in self-regulation and reward processing are modifiable via intervention and that mitigation of these deficits improves weight loss and weight maintenance outcomes.

In this review, we will first synthesize the literature on brain networks and obesity among adults. We will focus specifically on how functional network organization relates to obesity, as well as aberrant eating behaviors such as binge eating. We will then review what is known from longitudinal and clinical trial data regarding the effects of weight loss and specialized diets (e.g. the Mediterranean diet) on functional connectivity patterns. We seek to address the following questions: (i) How are functional brain networks that support self-regulation and reward processing associated with obesity and maladaptive eating behaviors? (ii) Can weight loss (via any means) modify obesity- and eating-related functional connectivity patterns? (iii) Are there specific nutrient or dietary patterns that are more effective than others for influencing brain connectivity patterns? This review focuses on evidence from adults with overweight and obesity, though it is important to note that there is a growing literature exploring these questions in children and adolescents (e.g. Chodkowski *et al.*, 2016; Liang *et al.*, 2014; Moreno-Lopez *et al.*, 2016) including prospective investigations of how regional brain activation relates to future weight gain (e.g. Yokum *et al.,* 2011). This research, which is critical to understanding the development of obesity and its impact on maturation, is beyond the scope of this review.

## Aberrant functional brain connectivity in obesity

### Task-evoked connectivity

The majority of fMRI studies of obesity have focused on isolated brain regions, although complex processes like self-regulation and reward valuation arise from interactions between brain regions. Functional connectivity is one method of capturing dynamic interactions between regions. Functional connectivity analyses provide a metric of how anatomically distinct brain regions are organized into coherent functional networks with specific properties (e.g. highly efficient local connections). This information can then be used to understand how variation in network organization affects behavioral processes supported by the network (Bullmore and Sporns, 2009; van den Heuvel and Pol, 2010). Moreover, characterizing how the functional organization of the brain differs or is altered in conditions like obesity may yield new insights about the neural mechanisms underlying these conditions. This may lead to improvements in treatment and prevention.

Obesity appears to be associated with variation in functional connectivity during the processing of food and monetary rewards, which may be related to altered self-regulation and reward processing. Indeed, several studies have observed that obesity is associated with increased connectivity between regions involved in valuation in the presence of reward cues, perhaps indicating that greater value is assigned to these cues among individuals with obesity. For example, obesity has been associated with stronger connectivity between the dorsal striatum, amygdala and insula (Nummenmaa *et al.*, 2012) and increased strength in connectivity between regions involved in salience detection such as the anterior insula and anterior cingulate cortex (ACC) (Kullmann *et al.*, 2013). Further, striatal connectivity to parahippocampal regions and the cerebellum in response to high calorie foods is greater in adults with obesity compared to healthy weight individuals (Carnell *et al.*, 2014), which may suggest that individuals with obesity are planning or imagining behaviors to obtain palatable food in response to relevant environmental cues. Finally, obesity has been associated with enhanced ventral striatal connectivity with the insula following monetary loss during a probabilistic learning task (Kube *et al.*, 2018). These results indicate that difficulty incorporating performance feedback into future decisions in obesity may be related to increased connectivity between regions involved in reward valuation and interoceptive awareness.

Interestingly, patterns of functional connectivity are modulated by hunger and satiation, which may be associated with approach motivation for high-calorie food cues. For instance, one study found that fasting was associated with enhanced connectivity between amygdala and ventral striatal seed regions and motor planning regions, particularly among men (Atalayer *et al.*, 2014). In contrast, participants exhibited greater functional connectivity between regions involved in salience detection and valuation such as amygdala, insula and striatum and regions involved in executive control and motor planning, including the ACC, supplementary motor area and dorsomedial prefrontal cortex (dmPFC) after consuming a full meal (Atalayer *et al.,* 2014). This finding may be interpreted as evidence that hunger enhances the reward value of food and promotes communication between emotion and reward processing regions and regions involved in executing movements, possibly to promote food intake, and that communication between regions supporting regulation and those involved in emotion and reward processing are strengthened when an individual is satiated. However, because this study did not include a normal weight comparison group, it is unclear if the observed patterns of functional connectivity are specific to obesity. Further, regions such as the ACC have complex functions that include aspects of both valuation and regulation, making it difficult to interpret which function is being engaged when an individual is processing high calorie food cues. Yet, these results suggest that hunger and satiation influence network-level signaling dynamics, which may underlie hunger-related enhancement of the reward value of food and the shift in motivation towards the immediate goal of alleviating hunger.

In contrast to the studies reporting increased connectivity between regions involved in stimulus valuation and motivational processes (Nummenmaa *et al.,* 2012; Kullmann *et al.,* 2013; Atalayer *et al.,* 2014; Carnell *et al.,* 2014), there are other studies that have observed obesity-related *reductions* in connectivity in these regions. However, cross-study heterogeneity in the conditions under which these patterns emerged may account for these discrepancies. One study applied an effective connectivity approach to determine whether obesity is associated with both the strength and the direction of communication between brain regions. This approach examines the direction of signals between regions, allowing inferences to be made regarding neural signal generated in one region on signal generated in another (Friston, 2011). Using this approach, effective connectivity from the amygdala to the nucleus accumbens (NAc) and orbitofrontal cortex (OFC) was weaker among adults with obesity compared to normal weight individuals when viewing high calorie foods after a fasting period (Stoeckel *et al.*, 2009). In contrast, the same study also demonstrated that obesity was related to *enhanced* effective connectivity between the OFC and NAc in response to viewing high-calorie foods (Stoeckel *et al.,* 2009). Reduced modulation of NAc and OFC signaling by the amygdala in response to food cues suggests that higher weight is associated with reduced signals between regions that are involved in supporting affective valence and incentive value, while increased OFC modulation of NAc is possibly indicative of greater subjective value being assigned to high calorie food cues (Stoeckel *et al.,* 2009). However, it will be necessary to evaluate these hypotheses by directly testing whether these patterns of effective connectivity in response to food cues predict affect- and reward-driven eating behavior. Obesity has also been associated with reduced connectivity between the amygdala and hippocampus, midbrain, thalamus and insula (Geha *et al.*, 2017), though this pattern emerged during the consumption of a milkshake rather than the more typical passive viewing of high-calorie food images while in a fasted state. Reduced signal coherence among these regions may reflect impaired updating and/or integration of homeostatic and motivational signals during the intake of high calorie food, which could contribute to overeating beyond caloric needs. However, given that calorie intake in the paradigm applied by Geha *et al.* (2017) was carefully titrated and not under participants’ volitional control, this interpretation is speculative. Further, as previously noted, hunger and satiation are associated with functional network organization in response to food cues, which may account for inconsistencies in the observed associations of obesity with indices of functional connectivity.

Another study demonstrated that connectivity strength between medial and lateral PFC and regions serving visual and motor functions was reduced in response to both food and monetary reward cues in obese relative to lean individuals (García-García *et al.*, 2013). This latter finding is consistent with the hypothesis that poor self-regulation and abnormal reward valuation often observed in obesity may be associated with weakened top-down neural communication from prefrontal regulatory regions to regions involved in planning and execution of motivated behaviors. Weaker connectivity strength between visual and motor regions, including extrastriate cortex, precuneus and primary motor cortex, and regions involved in inhibitory control like the IFG have also been observed in obesity (García-García *et al.,* 2013; Kullmann *et al.,* 2013; Geha *et al.,* 2017). This may reflect a relative disengagement of PFC-mediated modulation of visuospatial attention and motor output, which may promote biased attention to food and execution of behaviors to obtain food.

Frontostriatal and frontoparietal connectivity are also reduced in adults with obesity compared to normal weight individuals, which may be related to craving and eating of palatable food (Verdejo-Román *et al.*, 2017). For instance, weakened coupling within these networks was associated with greater willingness to pay for palatable food relative to less-appealing food of higher nutritional value, providing evidence that such network level impairments may be associated with real world decisions about which foods are purchased and consumed. Interestingly, successful regulation of craving, as well as successful response inhibition, are associated with the opposite pattern of cortical-subcortical connectivity among individuals with obesity, with stronger coupling being associated with more successful attenuation of craving and response inhibition (Tuulari *et al.*, 2015; Dietrich *et al*., 2016; Filbey and Yezhuvath, 2017). It is important to note that normal weight individuals did not exhibit enhanced connectivity between prefrontal and subcortical regions during successful craving regulation or response inhibition, suggesting that individuals with obesity might exhaust more cognitive resources when regulating craving and behavior. This may explain why some individuals experience frequent dietary self-regulation failures despite having the desire to engage in healthy behaviors. In general, findings from task-evoked connectivity analyses provide evidence that individuals with obesity exhibit differences in connectivity patterns in response to high calorie palatable food cues, which may underlie poor dietary self-regulation and weight gain.

### Resting-state connectivity

Although it is important to understand how differences in the functional organization of the brain during the presentation of food cues may relate to obesity, such studies do not answer whether such differences are limited to the processing of food cues or whether these areas are related to more domain-general processes (i.e. reward processing). Investigating functional network connectivity while the brain is not engaged in demanding tasks that require inhibitory control may afford insight into the ‘intrinsic’ functional organization of the brain (Fox and Raichle, 2007; Cole *et al.,* 2014). This information may then be used to draw inferences about obesity-related variations in brain function that might be more domain general or at least involved in several cognitive domains, including executive functioning, attention, self-regulation and reward processing.

Obesity has been associated with differences in functional connectivity at rest in many of the same regions and networks that are modulated by food cues described above. However, there is significant cross-study heterogeneity in the direction of the associations between obesity and resting network architecture that is likely attributable to the differences in the specific networks that emerge in each study, as well as differences in sample characteristics and approaches to quantifying resting connectivity (e.g. seed-based *vs* graph theory). Nevertheless, given what is known about how obesity is associated with processes such as self-regulation and reward valuation, it might be expected that obesity would be associated with weakened resting connectivity between brain regions that support self-regulation and those involved in valuation, but stronger connectivity between those involved in valuation. Indeed, several studies have observed this pattern among individuals with obesity (García-García *et al.*, 2013; Lips *et al.*, 2014; Wijngaarden *et al.*, 2015b). For instance, individuals with obesity have been shown to exhibit weaker amygdala connectivity with the inferior frontal gyrus (IFG) at rest (Lips *et al.*, 2014). Given the role of the IFG in response inhibition and attentional control (Hampshire *et al.*, 2010) and of the amygdala in valence processing (Davis and Whalen, 2001), it is possible that disrupted communication between these regions may reflect impairments in top-down control over attentional processing of cues in the environment. Interestingly, this pattern of weakened amygdala–IFG connectivity at rest was accompanied by stronger connectivity between the amygdala and insula (Lips *et al.,* 2014), which may underlie enhanced salience of motivational cues related to food. Similarly, obesity has been associated with stronger ventral striatal connectivity with ventromedial prefrontal cortex (vmPFC) (Contreras-Rodríguez *et al.*, 2017) and anterior cingulate cortex (ACC) at rest (Coveleskie *et al.*, 2015), which may lead to the overvaluation of food-related information that, in turn, leads to engagement in obesogenic behaviors. It is important to note that the attrition rate among individuals with obesity in Contreras-Rodriguez *et al.* (2017) was 28%, which may have produced biased results. Given this caveat, it will be necessary for future studies to replicate these findings.

There is evidence that obesity is associated with disruption of the default mode network (DMN), a canonical resting-state network that supports self-referential thinking, memory encoding and retrieval and social reasoning (Greicius *et al.*, 2003). For example, a study of weight discordant monozygotic twins observed enhanced connectivity of the DMN with regions located in other networks such as the occipital cortex among higher weight members of the twin pairs (Sadler *et al.,* 2018). Conversely, higher weight twins exhibited weakened connectivity in regions of the salience network (SN), including the ACC and anterior insula and the occipital pole compared to their lower weight sibling (Sadler *et al.,* 2018). These patterns of weight-dependent connectivity may be related to decreased sensitivity to homeostatic cues of hunger and satiety, as well as altered reward processing. Interestingly, there is evidence that functional connectivity *within* the DMN is reduced in obesity, a pattern that was accompanied by increased integration between DMN and other networks more centrally involved in processing externally generated stimuli (e.g. sensorimotor network; Doucet *et al.*, 2017). Similar patterns have been observed in other studies (Kullman *et al.*, 2012; García-García *et al.*, 2013; Geha *et al.,* 2017; Chao *et al.*, 2018), including a recent study examining the association of obesity with functional network organization in over 500 individuals (Beyer *et al.*, 2017). Disruption of the DMN in obesity may reflect a diminished capacity of this network to integrate information being generated by externally driven and internally driven processing, which may be related to impairments in attention, memory and meta-cognitive processes.

Some studies have documented obesity-related reductions in *global* connectivity strength, particularly between regions supporting self-regulation in the PFC and regions involved in stimulus valuation. For example, obesity has also been associated with decreased global and local efficiency, as well as modularity of functional networks throughout the brain (Baek *et al.*, 2017; Geha *et al.,* 2017; Chao *et al.,* 2018), suggesting that network architecture in obesity is characterized by reduced efficiency of information transfer both within and between networks and reduced functional segregation of networks. Moreover, there is evidence that some network hubs have reduced influence on neighboring regions in obesity, including the medial frontal gyrus (MFG; García-García *et al.*, 2015). The MFG is a region that has been implicated in cognitive processes disrupted in obesity, including motor planning, inhibitory control and conflict monitoring (Rushworth *et al.*, 2004). This indicates that the functional role of the MFG as a connective hub may be diminished in obesity. Intriguingly, modification of biased attention for high-calorie food cues through training increases MFG connectivity among individuals with obesity (Mehl *et al.*, 2019). This suggests that MFG connectivity may be associated with weight through variation in attentional control in the presence of highly salient motivational cues such as palatable food.

### Summary and limitations

There is growing evidence that obesity is associated with disruption of functional networks that are involved in cognitive, affective and behavioral self-regulation, as well as reward and interoceptive and homeostatic processes, which may be associated with weight-related impairments in these domains (see Table 1 for a summary of all studies of obesity). In particular, weight-related differences in connectivity have most frequently been noted in the executive control, salience and DMNs. Such global patterns in functional network organization may be related to many of the widespread cognitive and behavioral abnormalities that have been documented in obesity. Despite these advances in our understanding of the neural correlates of obesity, there remain a number of unresolved questions, the most important of which is the direction of causality. The majority of studies to examine the relationship between obesity and functional network organization have been cross-sectional, precluding any definitive statements regarding whether excess weight causes or is the consequence of disruption in these networks. Further, the direction of the relationship between obesity and indices of connectivity varies widely across studies, with some identifying patterns of weakened connectivity and others finding enhanced connectivity. Given that the behavioral consequences of network disruption in obesity are poorly understood, it is difficult to interpret the significance of these patterns of weakened or enhanced connectivity beyond what is known about the function of the affected networks or regions. Moreover, there have been limited efforts to replicate findings and there is significant heterogeneity in sample characteristics, connectivity techniques, and analytics approaches, making it difficult to evaluate the robustness of reported relationships. In order to translate these findings to improve treatment of obesity, additional research is necessary to better characterize the mechanistic pathways linking obesity with functional network disorganization.

**Table 1:** Summary of studies investigating the relationship between functional connectivity and obesity

**Author**	**Design**	**Variable of interest**	**FC technique**	***N***	**Attrition**	**Mean age (SD)**	**Mean BMI (SD)**	**Findings**
Chao *et al.,* 2018	Cross-sectional	Resting-state FC among individuals with obesity prior to bariatric surgery	Seed-based resting-state FC plus graph theory; the authors did not specify which seed(s) they used.	Obese: *n* = 20	n/a	Obese: 33.11 (8.86)	Obese: 37.66 (5.07)	Obesity was associated with increased FC in the bilateral anterior cingulate cortex and precuneus and with decreased FC in the medial prefrontal cortex. Obesity predicted lower cluster coefficients and modularity and higher global efficiency, suggesting a shift toward more random networks.
						Healthy weight: 45.03 (9.65)	Healthy weight: 22.64 (3.45)	
				Healthy weight: *n* = 30		*Significantly different		
Dietrich *et al.,* 2016	Cross-sectional	Relationship between obesity and FC during the regulation of food cravings; BMI was analyzed continuously.	PPI analysis during regulation of cravings	*N* = 43	n/a	26.7 (3.5)	27.5 (5.3)	BMI was positively associated with FC between the putamen and dorsal lateral and medial prefrontal cortex during regulation of craving.
García-García *et al.*, 2013	Cross-sectional	Functional activation and FC in response to high- and low-calorie food cues	ICA	Obese: *n* = 18	n/a	Obese: 34.78 (4.45)	Obese: 34.89 (4.78)	During the presentation of high-calorie food, obesity was associated with reduced FC of lateral and medial prefrontal regions and precuneus.
				Healthy weight: *n* = 19		Healthy weight: 32.00 (5.87)	Healthy weight: 22.44 (1.93)	
Kube *et al.,* 2018	Cross-sectional	Relationship of obesity with FC during a probabilistic learning task	PPI using the ventral striatum as the seed	Obese: *n* = 19	n/a	Obese: 29.5 (5.6)	Obese: 35.4 (4.5)	Individuals with obesity exhibited increased FC between the ventral striatum, insula and superior temporal gyrus during prediction error processing.
				Healthy weight: *n* = 23		Healthy weight: 30.0 (5.0)	Healthy weight: 22.5 (1.7)	
Sadler *et al.,* 2018	Cross-sectional	Effect of weight on resting-state FC in weight discordant identical twins	ICA	Weight discordant twins: *n* = 94	n/a	Weight discordant twins: 29.4 (3.5)	Weight discordant twins: 28.1 (5.6)	In BMI-discordant twins, twins with lower BMI had stronger FC between striatal/thalamic and prefrontal networks. FC patterns observed in the BMI-discordant twin sample were not seen in a BMI-similar sample, providing evidence that the results are specific to BMI discordance.
				Weight concordant twins: *n* = 94		Weight concordant twins: 28.5 (3.6)	Weight concordant twins: 25.6 (4.1)	
				Weight discordant unrelated individuals: *n* = 94		Weight discordant unrelated individuals: 29.3 (3.5)	Weight discordant unrelated individuals: 28.1 (5.7)	
Stoeckel *et al.,* 2009	Cross-sectional	Functional activation and FC in response to high- and low-calorie food cues	PPI using nucleus accumbens, amygdala and orbitofrontal cortex as seeds	Obese: *n* = 12	n/a	Obese: 27.8 (6.2)	Obese: 30.8–41.2	In response to high-calorie foods, individuals with obesity exhibited reduced amygdala FC with the orbitofrontal cortex and nucleus accumbens, but increased FC between orbitofrontal cortex and nucleus accumbens.
				Healthy weight: *n* = 12		Healthy weight: 28 (4.4)	Healthy weight: 19.7–24.5	
Verdejo-Román *et al.*, 2017	Cross-sectional	Characterize FC during processing of food and monetary rewards	Graph theory	Excess weight: *n* = 39	n/a	Excess weight: 33.59 (6.23)	Excess weight: 30.41 (3.69)	Excess weight was associated with decreased FC during the processing of food rewards in a network involving primarily frontal and striatal areas and increased FC during the processing of monetary rewards in a network involving principally frontal and parietal areas.
				Healthy weight: *n* = 37		Healthy weight: 33 (6.53)	Healthy weight: 22.28 (1.77)	
Lips *et al.,* 2014	Cross-sectional	Associations between fasting and resting-state FC among individuals with obesity with and without type II diabetes (T2DM)	Seed-based resting-state FC using the posterior cingulate cortex, hypothalamus and amygdala as seeds	T2DM: *n* = 19	n/a	T2DM: 47.7 (6.4)	T2DM: 43.8 (3.2)	No significant differences between normal-glucose-tolerant and T2DM subjects were observed. In the fasting state, obesity was associated with stronger hypothalamic FC with the medial prefrontal cortex and the dorsal striatum. The amygdala was differentially connected to the right insula in those with obesity. Food intake dampened hypothalamic FC with the frontal regions in lean subjects, whereas these connections were not affected in those with obesity.
				Non-T2DM: *n* = 27		Non-T2DM: 51 (7.1)	Non-T2DM: 42 (5.5)	
				Healthy weight: *n* = 12		Healthy weight: 49.2 (6.22)	Healthy weight: 21.7 (1.6)	
Wijngaarden *et al.,* 2015a	Cross-sectional	Associations between fasting and resting-state FC	Seed-based resting-state FC using the posterior cingulate cortex, hypothalamus and amygdala as seeds	Obese *n* = 13	n/a	Obese: 31 (3)	Obese: 35.4 (1.2)	At baseline, obesity was associated with stronger FC between hypothalamus and left insula. This effect diminished upon the prolonged fast.
				Healthy weight: *n* = 11		Healthy weight: 28 (3)	Healthy weight: 23.2 (0.5)	
Healthy weight: *n* = 31	Healthy weight: 27.05 (7.03)	Healthy Weight: 22.32 (19.52 to 25.09 kg m^2^)
		SD not reported
Coveleskie *et al.,* 2015	Cross-sectional	Associations between obesity and resting FC of the nucleus accumbens	Seed-based resting-state FC using the nucleus accumbens as a seed	Obese: *n* = 19	n/a	Obese: 25.42 (5.86);	Obese: 31.83 (25.88 to 37.56 kg m^2^)	Subjects with high BMI had greater FC of the left nucleus accumbens with bilateral anterior cingulate cortex and right ventromedial prefrontal cortex.
				Doucet *et al.,* 2017		Cross-sectional	Relationship between BMI and resting-state FC of canonical networks; BMI was analyzed continuously.		Graph theory	*N* = 496	n/a	29 (Range of 22–37 years)	26.6 (Range of 16.8–47.8 years)	Elevated BMI was associated with reduced functional cohesion and increased integration of sensory-driven networks (sensorimotor and visual) and internally guided networks (default mode and central executive).
				Beyer *et al.,* 2017		Cross-sectional	Relationship between BMI and resting-state FC of canonical networks; BMI was analyzed continuously.		ICA	*N* = 521	n/a	70.1 (3.8)	27.5 (4.1)	Higher BMI was significantly associated with lower default mode FC in the posterior cingulate cortex and precuneus.
Baek *et al.,* 2017	Cross-sectional	Relationship between obesity and resting-state FC	Resting-state FC between 90 regions defined using the AAL atlas	Obese: *n* = 20	n/a	Obese: 42.7 (11.1)	Obese: 33.4 (3.9)	Obesity was associated with global and local network efficiency as well as decreased modularity. In regional metrics, the putamen, pallidum and thalamus exhibited significantly decreased nodal degree and efficiency among individuals with obesity. Obesity was also associated with decreased FC of cortico-striatal/cortico-thalamic networks.
				BED: *n* = 20		BED: 43.7 (9.6)	BED: 33.0 (2.4)	
				Healthy weight: *n* = 40		Healthy weight: 41.8 (11.7)	Healthy weight: 22.5 (2.0)	
Garcia-Garcia *et al.*, 2015	Cross-sectional	Relationship between obesity and resting-state FC	Graph theory	Obese: *n* = 20	n/a	Obese: 33.55 (5.61)	Obese: 35.90 (5.83)	Individuals with obesity exhibited less degree centrality in the left middle frontal gyrus and the lateral occipital cortex.
				Healthy weight: *n* = 21		Healthy weight: 31.33 (5.96)	Healthy weight: 22.33 (1.87)	
Frank *et al.,* 2014	Cross-sectional	Effect of gastric bypass surgery on resting-state and task-evoked (food cue) FC	ICA	Obese: *n* = 11	n/a	Obese: 42.6 (4.0)	Obese: 40.2 (0.8)	Individuals with obesity who had not undergone surgery exhibited stronger FC in frontal regions of the DMN during resting state compared to healthy weight individuals and those who underwent surgery.
				Bypass surgery: *n* = 9		Bypass surgery: 42.0 (2.8)	Bypass surgery: 27.1 (0.9)	
				Healthy weight: *n* = 11		Healthy weight: 36.6 (3.8)	Healthy weight: 21.4 (0.5)	
Atalayer *et al.,* 2014	Cross-sectional	Sex differences in neural responsitivity to food cues	PPI analysis; seed-based FC on amygdala in response to high- *vs* low-calorie food cues in fed *vs* fasted state	Female: *n* = 14	n/a	Female: 35 (6.9)	Female: 36.9 (5.6)	In response to high- *vs* low-calorie food cues, obese men (*vs* women) had greater FC between amygdala and right subgenual anterior cingulate, whereas obese women had greater functional connectivity with amygdala in left angular gyrus and right primary motor areas.
				Male: *n* = 17		Male: 35 (9.0)	Male: 36.2 (5.5)	
Carnell *et al.,* 2014	Cross-sectional	Functional activation and FC in response to high and low calorie visual and auditory food cues	PPI analysis of a visual food cue task	Obese: *n* = 10	n/a	Obese: 22.4 (2)	Obese: 32.9 (5.3)	Individuals with obesity exhibited greater response to high-calorie cues, as well as relatively greater FC between the ventral tegmental area and cerebellum.
				Healthy weight: *n* = 10		Healthy weight: 21 (1.2)	Healthy weight: 22.1 (1.2)	
Geha *et al.,* 2017	Cross-sectional	Global brain FC during anticipation and tasting of a milkshake	Seed-based FC during the tasting of a milkshake	Healthy weight: *n* = 15	n/a	Obese: 27.7 (1.7)	Obese: 35.3 (.9)	At rest and during milkshake consumption, global FC was consistently decreased in the ventromedial and ventrolateral prefrontal cortex, insula and caudate nucleus and was increased in brain regions belonging to the dorsal attention network including premotor areas, superior parietal lobule and visual cortex.
				Obese: *n* = 15		Healthy weight: 27.4 (1.7)	Healthy weight: 21.9 (.5)	
Kullmann *et al.,* 2013	Cross-sectional	Relationship between obesity and FC during a visual food cue task	ICA	Obese/overweight: *n* = 12	n/a	Obese/overweight: 24.66 (2.42)	Obese/overweight: 30.46 (1.77)	Overweight/obesity was associated with increased FC of the salience network and networks involved in object recognition, motivational salience.
				Healthy weight: *n* = 12		Healthy weight: 22.91 (2.1)	Healthy weight: 21.16 (1.13)	
Nummenmaa *et al.,* 2012	Cross-sectional	Functional activation and FC in response to high- and low-calorie food cues	PPI analysis	Obese: *n* = 19	n/a	Obese: 45.72 (9.60)	Obese: 43.97 (3.74)	Obesity was associated with increased FC between the caudate nucleus and the amygdala, posterior insula and somatosensory cortex during the presentation of high calorie food.
				Healthy weight: *n* = 16		Healthy weight: 47.75 (10.44)	Healthy weight: 24.10 (2.07)	
Tuulari *et al.,* 2015	Cross-sectional	FC during craving regulation	PPI analysis	Obese: *n* = 27	n/a	Obese: 42.1 (9.3)	Obese: 41.4 (3.9)	Obesity was associated with higher FC in the executive control network during craving regulation.
				Healthy weight: *n* = 14		Healthy weight: 44.9 (11.9)	Healthy weight: 22.6 (2.7)	
Legget *et al.,* 2016	Longitudinal	Effects of a 6 month exercise program on resting-state FC	BNC with ICA	*N* = 11	9% (*N* = 1 did not complete post testing)	33.6 (1.4)	38.2 (3.2)	BNC in the posterior cingulate cortex was reduced following chronic exercise; change in BNC was related to changes in aerobic fitness level and perceived hunger.
Lepping *et al.,* 2015	Longitudinal	Resting-state FC after surgical and behavioral weight loss	Seed-based resting-state FC using mPFC, precuneus and inferior temporal gyrus	Bariatric surgery: *n* = 15	13% (*N* = 2 did not have useable follow-up data or failed to complete follow-up testing)	Bariatric surgery: 42 (10.35)	Bariatric surgery: 41.35 (1.97)	Following weight loss, behavioral dieters exhibited increased FC between left precuneus/superior parietal lobule and bilateral insula pre- to post-meal and bariatric patients exhibited decreased FC between these regions pre- to post-meal.
				Behavioral diet: *n* = 13		Behavioral diet: 40.23 (8.01)	Behavioral diet: 40.10 (1.8)	
Li *et al.*, 2018	Longitudinal	Resting-state FC among individuals with obesity who were awaiting bariatric surgery and a subset of that group who completed surgery	Resting-state FC between 90 regions defined using the AAL atlas	Preoperative: *n* = 29	24% (*N* = 7 surgery patients dropped out do to travel restrictions)	Preoperative: 27.8 (6.9)	Preoperative: 40.0 (6.5)	Pre-operative individuals exhibited increased FC in orbitofrontal cortex, MFG and superior frontal gyrus. Post-surgery, these differences were no longer present.
				Postoperative: *n* = 22 (subset of the preoperative group who underwent surgery)		Postoperative: Not described	Postoperative: 34.4 (5.9)	
				Healthy weight: *n* = 19		Healthy weight: 26.7 (6.8)	Healthy weight: 21.8 (1.8)	
Weygandt *et al.,* 2013	Longitudinal	FC during a dietary impulse healthy weight task as a predictor of weight loss	PPI using ventromedial prefrontal cortex as the seed	*N* = 16	NR	43.0 (12.2)	Pre-diet BMI: 34.5 (3.2)	Stronger FC between the dorsolateral and ventromedial prefrontal cortex was associated with better dietary success and impulse control.
							Post-diet BMI: 30.2 (1.8)	
Contreras-Rodríguez *et al.,* 2017	Longitudinal	Relationship between obesity and resting-state FC	Seed-based resting-state FC using the nucleus accumbens as a seed	Obese: *n* = 42	28% (*N* = 11 did not return to follow-up in the obese group)	Obese: 33.59 (6.16)	Obese: 30.51 (3.63)	Participants with excess weight displayed increased FC between the ventral striatum and the medial prefrontal and parietal cortices and between the dorsal striatum and the somatosensory cortex. Dorsal striatum FC correlated with food craving and predicted BMI gains.
				Healthy weight: *n* = 39		Healthy weight: 33.07 (6.73)	Healthy weight: 22.09 (1.74)	
McFadden *et al.,* 2013	Longitudinal	Effects of exercise on resting-state default mode and salience network activity in overweight/obese adults	ICA	*N* = *12*	0%	32.8 (9.5)	33.3 (4.3)	The intervention was associated with a reduction in DMN activity in the precuneus, which was associated with greater fat mass loss as well as reduced perceived hunger and hunger ratings in response to a meal. No changes were observed in the salience network in response to the exercise intervention.
Tregellas *et al.,* 2011	Randomized cross-over design comparing fed and fasted states	Effect of weight loss on FC of the DMN during the presentation of high calorie food cues	ICA	Reduced-obese: *n* = 18	NR	Reduced-obese: 35.2 (5.7)	Reduced-obese: 27.5 (2.6)	In the eucaloric state, greater activity among individuals who lost weight, compared to healthy weight individuals, was observed in the lateral inferior parietal and posterior cingulate cortices. Lateral parietal activity correlated positively with appetite. Overfeeding was associated with increased posterior cingulate default network activity in healthy weight individuals compared to those who lost weight.
				Healthy weight: *n* = 24		Healthy weight: 34.7 (5.4)	Healthy weight: 21.6 (1.7)	
Hinkle *et al.,* 2013	Randomized cross-over design comparing placebo *vs* leptin	FC of the hypothalamus during reduced-weight maintenance and leptin repletion	PPI analysis using the right hypothalamus as the seed	*N* = 10	NR	36.8 (6.5)	Initial: 39.9 (8.2)	During reduced-weight maintenance with placebo injections, the FC of the hypothalamus increased with visual areas and the dorsal anterior cingulate in response to food cues. During reduced-weight maintenance with leptin injections, FC of the right hypothalamus increased with the mid-insula and the central and parietal operculae, suggesting increased coupling with the interoceptive system and decreased with the orbital frontal cortex, frontal pole and the dorsal anterior cingulate.
							Following weight loss: 34.8 (7.0)	
Kahathuduwa *et al.,* 2018	RCT	Effect of two dietary manipulations on task-evoked FC	PPI analysis	Meal replacement (TMR) group: *n* = 16	12.5% (*N* = 4 did not complete study)	TMR: 31.27 (11.85)	TMR: 35.14 (3.75)	Compared to TD, TMR was also associated with negative modulation of FC of the nucleus accumbens, orbitofrontal cortex and amygdala by dorsolateral prefrontal cortex.
				Typical diet (TD) group: *n* = 16		TD: 32.15 (14.67)	TD: 34.82 (2.63)	
Filbey and Yezhuvath, 2017	RCT	Examine the relationship between BMI and successful inhibition during an inhibitory control task; BMI was analyzed continuously.	PPI analysis with IFG as the seed	*N* = 34	24% (*N* = 10 due to data quality; *N* = 1 due to BMI)	32.6 (10.6)	30.7 (6.3)	Positive correlations were found between BMI and impulsivity. Further, there was a positive association between BMI and FC between the right IFG and right middle frontal gyrus during successful response inhibition.
Mehl *et al.*, 2019	RCT	Task-evoked FC during cognitive bias modification training and resting-state FC after training	PPI analysis using the angular gyrus as a seed. Resting-state FC using dorsolateral and medial prefrontal cortex, amygdala, nucleus accumbens and MFG as seeds	Cognitive bias modification: *n* = 17	NR	Cognitive bias modification: 28(5)	Cognitive bias modification: 35.57 (4.63)	Analysis of brain FC during rest revealed training-related FC changes of the inferior frontal gyrus and bilateral middle frontal gyri.
				Healthy weight: *n* = 16		Healthy weight: 31 (4)	Healthy weight: 6.95 (7.63)	
						*Significantly different		

*Note.* BNC, between-network FC; PPI, psychophysiological interaction; FC, functional connectivity; ICA, independent component analysis; RCT, randomized controlled trial; NR, not reported.

## Longitudinal and intervention effects on the relationship between obesity and functional networks

### Task-evoked connectivity

Altered functional connectivity in response to palatable food cues may be related to intentional weight loss, with networks supporting self-regulation, reward processing and valuation and self-referent thought appearing to be most strongly related to alterations in weight. For example, individuals with overweight and obesity examined after recent weight loss exhibited greater connectivity in the DMN during passive viewing of food images compared to normal weight individuals (Tregellas *et al.*, 2011). Given the hypothesized role of the DMN in self-referent thought and internal monitoring, this may reflect a bias of attention to internal states such as hunger and craving (Tregellas *et al.,* 2011). These results suggest that altered DMN connectivity during the processing of food cues persists even after successful weight loss, though it is important to note that responses to food in the DMN prior to weight loss were not assessed in this study. It is possible that this pattern of connectivity may promote eating in response to food cues and may therefore underlie difficulty with weight maintenance after successful weight loss. Another study that examined the effect of two dietary interventions on functional connectivity evoked by food cues found that total meal replacement with reduced calorie shakes was associated with reduced dorsolateral prefrontal cortex (dlPFC) connectivity with NAc, amygdala, OFC and insula in response to food cues at post-intervention compared to pre-intervention among individuals with obesity (Kahathuduwa *et al.*, 2018). Interestingly, this effect was not observed with a standard reduced calorie diet typically administered for weight loss despite the fact that both diets prompted weight loss and reduced food cravings (Kahathuduwa *et al.,* 2018), indicating that meal replacement may exert a more robust effect on network organization. It is important to highlight that the effect of meal replacement on functional connectivity was evident after just 3 weeks of intervention (Kahathuduwa *et al.,* 2018), which suggests that dietary interventions may prompt rapid reorganization of networks involved in self-regulation and reward-related decision making.

Interestingly, there is experimental evidence that differences in functional connectivity during the processing of food cues that persist following weight loss is modified by leptin, a hormone released by adipose tissue that acts on hypothalamic receptors to signal weight status and regulate appetite (Frederich *et al.*, 1995; Maffei *et al.*, 1995). Specifically, administration of leptin during dietary weight loss reduced hypothalamic and NAc connectivity to the ACC, OFC and visual cortex but increased connectivity with the insula during the processing of high calorie foods (Hinkle *et al.*, 2013). This may indicate that leptin influences appetite and sensitivity to food cues through downstream effects on connectivity between regions involved in self-regulation, reward valuation and interoceptive functioning, and could be a useful adjunctive treatment in weight loss interventions if further research documents a relationship between leptin-induced changes in functional connectivity and weight-loss outcomes.

There is also evidence that patterns of connectivity prior to a weight loss attempt may be related to the amount of weight lost, with stronger connectivity between regions that support self-regulation being associated with greater weight loss. For example, among individuals with overweight and obesity, connectivity between dlPFC and vmPFC at baseline has been associated with the tendency to select the option to consume a larger portion of a preferred meal at a later time rather than a small portion immediately, despite being in a fasted state (Weygandt *et al.*, 2013). This finding suggests that enhanced connectivity between the dlPFC and vmPFC facilitates dietary impulse control, which is consistent with the role of these regions in self-regulation and in reward and emotion processing, respectively. Alternatively, this finding may also be interpreted as evidence that individuals who exhibit enhanced connectivity between the dlPFC and vmPFC simply assign more value to a higher calorie meal rather than possess more impulse control per se. However, this alternative explanation is weakened by the observation that stronger dlPFC–vmPFC connectivity and selection of larger delayed meals at baseline predicted weight loss following a dietary intervention (Weygandt *et al.,* 2013). Two tentative conclusions can be drawn from these studies. First, the findings reported by Tregellas *et al.* (2011) and Hinkle *et al.* (2013) suggest that some obesity-related differences in network-level connectivity may not be attenuated by weight loss. It is possible, therefore, that certain patterns of functional connectivity in obesity may represent a stable marker of the condition that may either increase risk for obesity or facilitate maintenance of unhealthy weight. Alternatively, these differences in functional connectivity may potentially increase vulnerability for weight gain after a successful reduction in weight. Second, given that patterns of connectivity associated with enhanced impulse control predict the amount of weight lost following a dietary intervention (Weygandt *et al.,* 2013), it may be argued that network-level connectivity patterns are mechanistically linked to obesity through processes like self-regulation and reward valuation and may therefore precede the development of obesity. However, it will be necessary to compare connectivity patterns before and after weight loss and stable weight maintenance, as well as to measure connectivity patterns in individuals at high risk for obesity, to evaluate these hypotheses. See Table 1 for a summary of these studies.

### Resting-state connectivity

Several recent studies also provide prospective and intervention-related evidence that resting-state functional connectivity patterns both predict future weight gain and are modified by weight loss, suggesting that differences in resting-state functional connectivity may be mechanistically linked to obesity. For instance, stronger dorsal striatal connectivity with the somatosensory cortex at baseline was found to predict additional weight gain at 12-week follow-up among individuals with obesity (Contreras-Rodriguez *et al.*, 2017), suggesting that enhanced communication between regions involved in sensory processes and those involved planning and execution of motivated behaviors may promote engagement in behaviors that lead to weight gain. However, as noted above, these findings should be interpreted with caution given the high rate (28%) of participant drop-out in this study. Cross-sectional comparisons between normal-weight individuals, individuals with current obesity and individuals who underwent gastric bypass surgery at least 1 year prior to enrollment revealed that connectivity between the ACC, OFC and superior frontal gyrus was greater among individuals with obesity compared to normal-weight individuals and those who had undergone bariatric surgery (Frank *et al.*, 2014). A similar pattern was observed for resting-state connectivity between regions involved with reward and somatosensory processing (e.g. OFC and post-central gyrus), such that individuals with obesity exhibited heightened connectivity compared to normal-weight individuals and individuals with obesity who had lost weight following surgery (Li *et al.*, 2018b). Further, there were no significant differences in connectivity strength between the normal weight and surgery groups (Frank *et al.,* 2014; Li *et al.*, 2018a). As such, it is possible that weight loss may induce changes in functional connectivity that promote normalization of reward valuation and enhance self-regulation. However, this interpretation is speculative given that these studies did not assess baseline resting-state connectivity or examine the relationship between resting-state connectivity and cognitive and behavioral risk factors for obesity, including reward processing, impulsivity or diet. Therefore, it is currently unknown whether weight loss-induced variation in resting-state connectivity relates to individual differences in cognitive and behavioral processes that could influence the extent of post-intervention weight regain. Additional research is necessary to better understand how obesity and weight loss affect resting-state connectivity and whether differences in resting-state connectivity are associated with cognitive or behavioral risk factors for obesity. Further, it will be important to determine whether individual differences in resting-state connectivity following weight loss predicts the extent of weight regain after an intervention is complete.

Data from intervention and longitudinal studies provide further support for the hypothesis that weight loss produces changes in resting-state connectivity, particularly in the DMN. Specifically, while evidence suggests that obesity is associated with stronger DMN connectivity at rest (e.g. Sadler *et al.,* 2018), weight loss has consistently been associated with reductions in DMN connectivity. For instance, following a 6 month physical activity intervention, participants exhibited reduced connectivity between the precuneus and other regions comprising the DMN relative to baseline (McFadden *et al.*, 2013). Similar results were reported by this group using data drawn from the same sample but applying an effective connectivity approach to quantify intervention related changes (Legget *et al.*, 2016). Further, greater reduction in DMN connectivity strength over the course of the intervention predicted greater reduction in fat mass and hunger ratings during and after a meal (McFadden *et al.,* 2013), indicating that changes in connectivity may underlie improved behavioral self-regulation in the service of meeting weight loss goals. Bariatric surgery has similarly been shown to weaken resting connectivity between nodes of the DMN, including the PCC, hippocampus and vmPFC, with the extent of this reduction in connectivity strength being associated with greater weight loss following surgery (Li *et al*., 2018a). Individuals who have undergone bariatric surgery also exhibit reduced connectivity between regions involved in value assignment and motivational processes such as the vmPFC, dmPFC and ACC (Li *et al.*, 2018b), which may promote healthy lifestyle behaviors necessary to maintain weight loss following surgery.

Resting-state connectivity patterns in the DMN may also vary according to the method of weight loss, suggesting that some interventions may be more effective at modifying network function to support weight loss efforts. Specifically, cross-sectional assessment of individuals who participated in a weight loss intervention demonstrated that those who underwent gastric bypass surgery exhibited increased superior parietal lobe connectivity with the insula, superior temporal lobe and primary motor cortex during a fasted state compared to individuals who participated in a dietary restriction program (Lepping *et al.*, 2015). In addition, connectivity between these regions weakened following the administration of a meal among gastric bypass surgery patients, but not among individuals in the dietary intervention (Lepping *et al.,* 2015). This pattern of results may be interpreted as evidence that behavioral weight loss interventions attenuate biases in attention to signals of hunger and promote awareness of satiety signals, an effect that may be absent in surgical interventions due to their limited focus on changing dietary-related cognitions and behaviors (Lepping *et al.,* 2015). It is possible that differences in DMN connectivity across intervention methods may influence the degree to which each intervention produces changes in dietary behavior and weight. However, this study did not examine whether differences in connectivity either before or after meal administration were associated with degree of weight lost during the intervention, with dietary behaviors outside of the laboratory, or with long-term maintenance of lowered weight. Further, they were unable to examine changes in connectivity from baseline to post-intervention time points, information that could prove useful for understanding mechanisms influencing treatment response. Therefore, it is as of yet unclear whether differences in resting-state connectivity between weight loss intervention groups are related to the long-term efficacy of each intervention, an interesting question warranting additional research. These studies provide preliminary evidence that weight loss leads to changes in resting-state connectivity that may facilitate or mediate improved control of diet and physical activity. Moreover, they suggest that altered resting-state connectivity patterns may be one mechanism by which obesity develops and/or is maintained (see Table 1).

## Relationship between disordered eating behaviors and functional connectivity

The prevalence of eating psychopathology characterized by binge eating [e.g. bulimia nervosa (BN) and BED] is elevated among individuals with obesity (Kessler *et al.*, 2013) and is associated with the use of ineffective dieting strategies (e.g. dietary restraint; Klesges *et al.,* 1992). Moreover, rates of obesity among individuals diagnosed with BN and BED significantly exceed that of the general population (Villarejo *et al.,* 2012). Therefore, examining the relationship between functional network organization and clinical eating phenotypes such as binge eating and dietary restraint may yield additional insights regarding the behavioral, psychological and neural correlates of obesity. To date, only 11 studies have examined the relationship between functional network organization and clinical eating phenotypes most typically associated with obesity. Of these, only two studies included individuals with a BMI in the overweight or obese range (Geliebter *et al.*, 2016; Stopyra *et al.*, 2019). Further, very little is known about functional network organization in BED, the eating disorder most frequently co-occurring with obesity. Nevertheless, these studies may serve as a basis for additional research exploring the relationship between functional network characteristics and disordered eating among individuals with overweight and obesity. A summary of these studies is provided in Table 2.

**Table 2:** Summary of studies investigating the relationship between FC, disordered eating and diet

**Author**	**Design**	**Variable of interest**	**FC technique**	***N***	**Attrition**	**Mean age (SD)**	**Mean BMI (SD)**	**Findings**
Chen *et al.,* 2016	Cross-sectional	Eating disorder pathology and FC of the dorsolateral prefrontal cortex	Seed-based resting-state FC; voxel-mirrored homotopic resting-state FC	Restrained eaters: *n* = 23; healthy controls: *n* = 24	n/a	Restrained eaters: 20.74 (1.51); healthy controls: 21.04 (1.85)	Restrained eaters: 21.09 (2.38); controls: 21.01 (2.50)	Restrained eaters exhibited reduced interhemispheric connectivity of the dorsolateral prefrontal cortex, which was associated with higher BN symptoms. Increased dorsolateral prefrontal cortex resting-state connectivity was associated with BN pathology but only among restrained eaters.
Lee *et al.,* 2014	Cross-sectional	The relationship between eating disorder diagnosis and resting-state FC of the dorsal anterior cingulate cortex	Seed-based resting-state FC of the dorsal anterior cingulate cortex	AN: *n* = 18; BN: *n* = 20; healthy controls: *n* = 20	n/a	AN: 25.2 (4.2); BN: 22.9 (3.9); healthy controls: 23.3 (1.8)	AN: 16.0 (1.7); BN: 21.6 (2.3); healthy controls: 19.9 (1.9)	AN group exhibited stronger synchronous activity between the dorsal anterior cingulate cortex and retrosplenial cortex, whereas the BN group showed stronger synchronous activity between the dorsal anterior cingulate cortex and medial orbitofrontal cortex. Both groups demonstrated stronger synchronous activity between the dorsal anterior cingulate cortex and precuneus, which correlated with higher scores of the Body Shape Questionnaire.
Lavagnino *et al.,* 2014	Cross-sectional	The relationship between BN diagnosis and FC of salience, executive control, somatosensory, DMNs	Seed-based resting-state FC using a single node from each network of interest as a seed	BN: *n* = 16; healthy controls: *n* = 18	n/a	BN: 23 (5); healthy controls: 23 (3)	BN: 21 (2); healthy controls: 22 (2)	BN group showed a decreased resting FC both within the somatosensory network and with posterior cingulate cortex and two visual areas (the right middle occipital gyrus and the right cuneus).
Wang *et al.,* 2017	Cross-sectional	Relationship between resting-state connectivity assessed via graph theory and BN diagnosis	Resting-state connectivity assessed via graph theory	BN: *n* = 44; healthy controls: *n* = 44	n/a	BN: 22 (3.4); healthy controls: 23.1 (3.4)	BN: 21.0 (2.6); healthy controls: 20.5 (1.4)	Nodal strength in BN was higher in the sensorimotor and visual regions as well as the precuneus, but lower in several subcortical regions, such as the hippocampus, parahippocampal gyrus and orbitofrontal cortex. BN group also showed hypoconnectivity involving subcortical limbic and paralimbic regions, which correlated significantly with scores of bulimia and drive for thinness.
Stopyra *et al.,* 2019	Cross-sectional	Relationship between resting-state connectivity and eating disorder diagnosis	Seed-based resting-state connectivity	BED: *n* = 27; BN: *n* = 29; healthy weight controls *n* = 29; overweight controls: *n* = 28	n/a	BED: 38.39 (13.06); BN: 27.45 (10.55); healthy weight controls: 26.86 (6.59); overweight controls: 39.40 (10.48)	BED: 32.64 (4.13); BN: 21.33 (2.99); healthy weight controls: 21.85 (1.80); overweight controls: 33.58 (4.54)	ED patients exhibited aberrant FC in the dorsal anterior cingulate cortex within the salience network, as well as in the medial prefrontal cortex. Furthermore, BED and BN groups differed from each other in FC within each network. Seed-based analysis revealed stronger synchronous dorsal anterior cingulate cortex-retrosplenial cortex activity in the BN group.
Canna *et al.,* 2017	Cross-sectional	Resting-state VMHC	VMHC	AN: *n* = 15; BN: *n* = 13; healthy controls: *n* = *1*6	n/a	AN: 25.3 (1.6); BN: 27.2 (2.0); healthy controls: 26.1 (3.5)	AN: 16.8 (1.6); BN: not reported; healthy controls: 21.1 (1.6)	Compared to HC, AN patients exhibited reduced VMHC in cerebellum, insula and precuneus, while BN patients showed reduced VMHC in dorsolateral prefrontal and orbitofrontal cortices.
Frank *et al.,* 2016	Cross-sectional	Effective connectivity between regions involved in energy homeostasis and reward during the ingestion of a sucrose solution among individuals with an ED diagnosis	Effective connectivity	AN: *n* = 26; BN: *n* = 25; healthy controls: *n* = 26	n/a	AN: 23.23 (5.26); BN: 24.64 (3.22); healthy controls: 24.39 (3.49)	AN: 16.23 (1.09); BN: 23.56 (5.89); healthy controls: 21.61 (1.21)	Only the controls had an effective connectivity pattern from the hypothalamus to ventral striatum bilaterally. On the right side both eating-disorder groups showed effective connectivity from the anterior cingulate to ventral striatum and from there to the hypothalamus. Both AN and BN showed effective connectivity on the left from ventral anterior insula to inferior orbitofrontal cortex, middle to inferior orbitofrontal cortex and dorsal to ventral anterior insula.
Geliebter *et al.,* 2016	Cross-sectional	Task-evoked FC of the dorsal anterior cingulate cortex in response to food cues among individuals who binge eat	Seed-based connectivity, PPI	Binge eating: *n* = 10; healthy controls: *n* = 10	n/a	Binge eating: 22.1 (2.3); healthy controls: 21.3 (0.6)	Binge eating: 27.4 (5.8); healthy controls: 27.7 (7.2)	In response to high-calorie (*vs* low-calorie) food cues, binge eaters exhibited greater connectivity between dorsal anterior cingulate cortex and insula, cerebellum and supramarginal gyrus. None of these effects were significant at the whole-brain level. No effects of body weight were observed.
Kim *et al.*, 2012	Cross-sectional	Task-evoked functional and effective connectivity of the anterior insula during the processing of food cues	Seed-based connectivity, PPI with anterior insula as the region on interest	AN: *n* = 18; BN: *n* = 20; healthy controls: *n* = 20	n/a	AN: 25.2 (4.2); BN: 22.9 (3.9); healthy controls: 23.3 (1.8)	AN: 16.0 (1.7); BN: 21.6 (2.3); healthy controls: 19.9 (1.9)	In response to food images compared to non-food images, both the AN group and BN group demonstrated increased activity in the left anterior insula. In the AN group, the left anterior insula demonstrated significant interactions with the right insula and right IFG. In the BN group, the left anterior insula demonstrated significant interactions with the medial orbitofrontal cortex.
Bohon and Stice, 2012	Cross-sectional	Task-evoked connectivity during anticipation and receipt of a chocolate milkshake	Seed-based connectivity, PPI with amygdala as the ROI	Subthreshold BN: *n* = 13; healthy controls: *n* = 13	n/a	20.3 (1.87); subgroup means not reported	BN: 23.93 (2.82); healthy controls: 23.19 (2.42)	Connectivity analyses revealed a greater relation of amygdala activity to activation in the left putamen and insula during anticipated receipt of milkshake in the BN group relative to the control group. The opposite pattern was found for the taste of milkshake.
Neveu *et al.,* 2018	Cross-sectional	FC of dorsolateral prefrontal cortex during a food choice task	Seed-based PPI with dorsolateral prefrontal cortex as the ROI	BN: *n* = 35; healthy controls: *n* = 26	n/a	BN: 24 (3.87); healthy controls: 23 (2.70)	BN: 19.9 (2.15); healthy controls: 21.3 (2.36)	BN patients chose unhealthy food more often. FC analysis showed that the activity in the dorsolateral prefrontal cortex was coupled with ventromedial prefrontal cortex activity in uncontrolled food choices.
**Diet and FC studies (humans)**								
Garcia-Casares *et al.*, 2017	Longitudinal intervention (no control group)	Resting-state seed-based connectivity	Seed-based	*N* = 19	15.8% (*N* = 3 did not complete follow-up)	46.31 (4.07)	38.15 (4.7)	After the intervention, there was decreased FC between the left inferior parietal cortex and the right temporal cortex, and bilateral posterior cingulate; decreased connectivity between the left superior frontal gyrus and the right temporal cortex; decreased connectivity between the prefrontal cortex and the somatosensory cortex; and decreased connectivity between the left and right posterior cingulate.
Talukdar *et al.*, 2019	Cross-sectional	Whole-brain connectivity	Multivariate distance-based matrix regression	*N* = 96	n/a	69.0 (3.0)	26.0 (4.0)	Omega 3 levels were associated with individual differences in FC within regions that support executive function (prefrontal cortex), memory (hippocampus) and emotion (amygdala).

*Note.* ED, eating disorder; PPI, psychophysiological interaction; FC, functional connectivity; VMHC, voxel-mirrored homotopic connectivity.

### Task-evoked connectivity

Five of the 11 studies to examine functional network characteristics among individuals who engage in binge eating have interrogated whether and how functional networks are engaged in the presence of food-related stimuli. Several of these studies have identified differences in connectivity of nodes in the SN. For instance, participants with subthreshold BED exhibited increased dorsal ACC connectivity with the anterior insula, cerebellum and supramarginal gyrus during visual and auditory presentation of high-calorie food cues compared with participants who did not endorse binge eating (Geliebter *et al.,* 2016). These regions have been implicated in affective and interoceptive awareness, processes that allow the use of homeostatic and motivational states to guide decision making (Craig, 2009; Shenhav *et al.,* 2013), suggesting that disrupted connectivity between these regions may contribute to binge eating through impaired self-awareness of affect, arousal and hunger. Individuals with BN, a disorder that includes recurrent episodes of binge eating accompanied by compensatory behaviors intended to mitigate weight gain, were found to exhibit increased connectivity between the anterior insula and the medial OFC in response to high calorie-food cues (Kim *et al.,* 2014). This pattern was not present among those diagnosed with anorexia nervosa (AN; Kim *et al.,* 2014), an eating disorder in which binge eating is more rarely observed and is instead characterized by extreme caloric restriction and low body weight. This may indicate that increased coupling of the insula and OFC during the processing of high-calorie food cues is a distinct feature of binge eating. Similarly, another study found that subthreshold BN was associated with increased amygdala connectivity with the insula and putamen during anticipation of tasting a milkshake, highlighting the importance of the insula in binge eating pathology (Bohon and Stice, 2012). Finally, greater connectivity between the dlPFC and vmPFC was associated with more frequent selection of unhealthy calorie dense foods among individuals with BN, a relationship that was not apparent among healthy individuals (Neveu *et al.*, 2018). Together, these findings suggest that binge eating psychopathology is associated with differences in network organization and regions that support reward valuation and salience detection during the processing of food cues.

There is also evidence that effective connectivity between regions involved in reward processing and salience detection is altered in eating disorders. Interestingly, one study found that individuals without an eating disorder showed heightened effective connectivity from the hypothalamus to the ventral striatum while tasting a sucrose solution (Frank *et al.*, 2016). In contrast, communication between these two regions was shown to flow in the opposing direction among individuals with AN and BN, with the ventral striatum exerting influence over the hypothalamus (Frank *et al.,* 2016). Individuals with BN exhibited a unique pattern of heightened effective connectivity from the ACC to medial OFC in response to sucrose (Frank *et al.,* 2016). These results suggest that eating disorder psychopathology not only is associated with differences in signal coherence between regions involved in reward and salience detection but also may alter the direction in which signals are propagated.

### Resting-state connectivity

The majority (*n* = 6) of studies to investigate variation in functional connectivity among individuals with binge eating psychopathology have focused on intrinsic functional networks. BN has been associated with differences in resting-state networks that support self-referential processing, detection of and orientation toward salient cues in the environment, assignment of value to experience and somatosensory processes, including the SN, the somatosensory network, and the DMN. For instance, individuals with BN exhibited stronger nodal strength of regions in somatosensory and occipital cortex, as well as the precuneus, compared to those without an eating disorder (Wang *et al.*, 2017). This suggests that regions involved in body awareness and sensation might be more integrated in BN, which may be associated with body image distortions and enhanced responsiveness to food cues. Similarly, BED has also been linked to enhanced resting connectivity between regions of somatosensory cortex (Stopyra *et al.,* 2019). Moreover, the strength of connectivity was positively correlated with the frequency of binge episodes (Stopyra *et al.,* 2019), providing evidence that stronger coupling of these regions represents a neural signature of binge eating. However, it is important to note that another investigation of intrinsic connectivity of somatosensory regions observed reduced rather than enhanced coherence among individuals with BN (Lavagnino *et al.*, 2014), which presents some interpretive difficulties regarding the role of these regions in binge eating.

Nodal strength has been shown to be altered in regions that are critical for salience detection, reward and affective processes and memory, including the OFC, striatum, putamen, amygdala, insula and hippocampus (Wang *et al.,* 2017). This finding is consistent with other studies demonstrating that binge eating phenotypes are associated with differences in the SN at rest, particularly the dorsal ACC and anterior insula (Stopyra *et al.,* 2019; Lee *et al.*, 2014). For instance, women with BN were shown to exhibit enhanced connectivity between the dorsal ACC and medial OFC, a pattern that was not observed in AN (Lee *et al.,* 2014). Moreover, stronger resting connectivity between the dorsal ACC and precuneus was related to more severe weight and shape concerns among both eating disorder subgroups (Lee *et al.,* 2014), suggesting that disruptions in the SN and DMN may be associated with body image distortions common to both diagnoses. Connectivity of the dlPFC has also been shown to be altered among individuals high in dietary restraint, a common transdiagnostic feature of eating disorders. Specifically, dietary restraint was associated with reductions in connectivity between the dlPFC and regions involved in reward processing (e.g. vmPFC) and self-directed thinking and memory (PCC; Chen *et al.*, 2016), which may contribute to episodes of disinhibited eating frequently experienced by individuals high in dietary restraint. This hypothesis is supported by the observation that reductions in dlPFC connectivity were associated with symptoms of BN among those characterized as restrained eaters (Chen *et al.,* 2016). Finally, interhemispheric connectivity of the dlPFC and the OFC has been shown to be diminished in BN (Canna *et al.*, 2017), providing further evidence that networks supporting cognitive control and reward processes are disrupted in conditions such as BN.

### Summary and limitations

Overall, there have only been 11 studies to investigate the relationship between functional connectivity and clinical eating phenotypes most commonly documented in obesity **(**Table 2). Nevertheless, these studies have identified a number of networks that may be disrupted among individuals with eating psychopathology, particularly the salience, somatosensory, reward and DMNs. However, because only two of the studies described above included individuals who were overweight or obese (Geliebter *et al.,* 2016; Stopyra *et al.,* 2019), it is difficult to draw inferences based on these findings regarding eating psychopathology and network connectivity specifically in this population. Additional research examining whether the relationship between eating psychopathology and functional network organization is modified by weight status or moderates the efficacy of weight loss interventions is warranted.

## Relationship between nutrient intake profiles and functional connectivity

The evidence summarized in the sections above suggests that it is not only the state of obesity but also specific eating behaviors (e.g. binge eating) that are related to functional connectivity networks. This raises the question as to whether there are certain nutrients or macronutrient intake profiles (i.e. diets) that are associated with functional connectivity and cognitive health. Such information could lead to mechanistic research examining personalized dietary interventions for individuals with obesity that are designed to target the most disrupted brain networks.

Accumulating evidence suggests that one particular nutrient family, long-chain polyunsaturated omega 3 fatty acids (ω-3FAs), specifically docosahexaenoic acid (DHA) and eicosapentaenoic acid (EPA), are associated with both structural and functional brain health. In fact, one of these fatty acids, DHA, is the principle ω-3FA comprising brain gray matter (Calder, 2016), so there is good reason to suspect that supplementing concentrations of ω-3FAs would affect the functioning of brain networks. As with most intervention research, much of the initial groundwork regarding the effects of specific nutrients or entire diets on functional connectivity has been conducted using animal models. Studies of functional connectivity using animal models almost exclusively examine resting-state connectivity patterns for practical reasons (i.e. because animals are typically sedated in order to undergo fMRI).

### Diet and resting-state networks in animals

Using a macaque model, Grayson *et al.* (2014) examined the effects of a ω-3FA-deficient diet on functional brain development in monkeys that had been placed on control diets providing DHA or a non-DHA ω-3FA, or to an experimental group providing no ω-3FAs (‘ω-3FA-deficient’) in early life. Monkeys that were raised on the ω-3FA-deficient diet demonstrated much weaker connectivity between nodes in the early visual pathway compared to the other groups. The deficient monkeys also had weaker, less segregated connectivity within higher-order association networks thought to support executive functioning in humans, as well as reduced global efficiency across brain networks. These functional connectivity differences were linked to behavioral deficits in visual acuity and attention in the ω-3FA-deficient monkeys compared to those consuming ω-3FAs (Reisbick *et al.*, 1994). Importantly, monkeys consuming the non-DHA ω-3FA diet had intermediary connectivity strength patterns to those fed the DHA-rich diet. These results not only demonstrate a sustained effect of ω-3FA deficiency on functional connectivity but also provide evidence supporting that ω-3FA supplementation (esp. with DHA) relates to more developmentally favorable functional connectivity patterns in brain networks supporting both basic (i.e. visual) and higher-order (sustained attention) cognitive processes. Thus, nutrient intake profiles higher in ω-3FA may optimize the development and ultimate functioning of key brain networks supporting both higher order and basic functions.

Importantly, the evidence for the positive effects of ω-3FA supplementation does not appear to be limited to early life. A study by Weismann *et al.* (2016) demonstrated that DHA supplementation can have beneficial effects in older animals exhibiting signs of pathological aging. Older mice carrying the APOE-e4 allele, a genetic risk factor for Alzheimer’s disease, were fed a diet enriched with vitamins and DHA. Importantly, these mice were exhibiting structural and functional signs of Alzheimer’s disease, including weakened functional connectivity in several age-vulnerable regions (e.g. the hippocampus) and histological profiles indicating neural distress. Compared to a group of APOE-e4 mice fed with a typical diet, those fed with the enhanced diet showed increased hippocampal and visual cortex functional connectivity. In fact, their functional connectivity networks were more similar to mice not carrying the APOE-e4 allele. Thus, supplementation of a diet rich in ω-3FAs may be able to ‘rescue’ aberrant functional connectivity patterns associated with disease and delay or reverse neural changes associated with pathological aging. Intriguingly, some of the same regions most sensitive to the effects of aging and ω-3FA supplementation (e.g. the hippocampus and sensory regions) are also among those implicated in obesity in humans, suggesting some degree of shared mechanisms between weight regulation, eating behaviors and aging.

### Diet and resting-state networks in humans

Although the positive effects of ω-3FA supplementation on cognitive and brain structural development in humans is well established (for review see Lauritzen *et al.,* 2016), evidence for its effects on brain functional connectivity specifically is more sparse. However, a growing number of human resting-state fMRI studies are reporting findings consistent with the animal studies of ω-3FA rich diets summarized above.

García-Casares *et al.* (2017) examined the effects of a 6 month weight loss intervention involving Mediterranean diet (a diet rich in ω-3FAs) and physical activity on functional connectivity in adult women with obesity. All individuals in the sample successfully lost weight during the intervention. Further, at follow-up, connectivity between regions involved in networks affected by obesity (e.g. DMN, salience, orexigenic and sensory motor networks) was altered. These results were interpreted as showing a normalizing functional connectivity pattern in a sample with obesity following successful weight loss. However, there were several notable limitations of this study that make firm conclusions tenuous. First, the study did not include a control group, and so it is not possible to tell whether functional connectivity patterns became more similar to healthy-weight females, or just changed more generally. Secondly, it is not possible to isolate the effects of Mediterranean diet from PA or caloric restriction in this study since, in addition to partaking in a Mediterranean diet, participants were also encouraged to increase their PA levels and decrease their caloric intake.

Fortunately, the results of other studies have more firmly linked differences in functional connectivity patterns to ω-3FA acid intake. In a cross-sectional study of 96 older adults, Talukdar *et al.* (2019) conducted a comprehensive connectome-wide study on which functional connections are associated with individual differences in ω-3FA levels. They found that ω-3FAs are associated with increased functional connectivity within frontal lobe regions underlying executive functioning, as well as among temporal lobe regions involved in memory and emotional salience. Further, the strength of these connections was associated with measures of intelligence. These results suggest that certain brain connections are sensitive to ω-3FA levels and are associated with cognitive operations (i.e. intelligence) linked to executive functioning, attention and memory.

### Limitations of human work on diet and functional connectivity

To date, most of the work linking specific diets or dietary supplements to functional connectivity have been cross sectional in nature, and those with multiple time points have not included a control group. This is likely for practical reasons given the difficulty of controlling diet in humans. Nonetheless, randomized controlled trials will be important to make causal links between diet, or specific nutrients, and functional connectivity. Further, there have been no studies to examine the effect of diet on task-evoked functional connectivity in adults. Although resting-state connectivity patterns have been shown to be behaviorally meaningful, it is also important to understand how diet might modulate task-evoked connectivity patterns, particularly during tasks in which individuals with obesity or disordered eating show behavioral performance deficits. Finally, the majority of work on relating diet to functional connectivity is Omega-3 centric. However, there are other popular diets, such as the ketogenic diet, that are already key therapeutic tools in other clinical contexts, such as epilepsy (Henderson *et al.,* 2006; Lutas and Yellen, 2013) and are emerging as effective for weight loss in the larger wellness industry (Masood and Uppaluri, 2019). However, to our knowledge, no studies have systematically examined the relationship of this or other diets to functional brain health in the context of obesity. Relatedly, there have been no studies to examine the effect of problematic dietary intake patterns such as the consumption of ultra-processed foods on functional network characteristics, an important future direction for research given evidence that these foods are becoming the predominant global food source (Monteiro *et al.*, 2013) and have been shown to be particularly detrimental to health (Hall *et al.*, 2019; Monteiro *et al.*, 2013).

## Summary and conclusions

There is strong evidence to suggest that obesity is associated with differences in network signaling dynamics, both in the context of specific task demands (e.g. valuation of food cues) and at rest and that these signaling variations may relate to variation in weight because they are involved in supporting self-regulation and reward processing. Further, there is emerging evidence that disruptions of networks involved in salience detection, self-referential thought, reward processing and executive control may be related to eating patterns that promote obesity. Fortunately, data drawn from the scant but growing weight loss intervention literature suggests that these patterns of functional connectivity are modifiable and may facilitate weight loss through improvements in self-regulation and dietary decision making. These findings provide a more complete understanding of the neural correlates of obesity and suggest that functional brain patterns might predict eating behaviors but might also be affected by weight gain.

Despite these advances, there remain a number of open questions that warrant additional research. First, the relationship between obesity and functional connectivity is highly complex, with there being evidence of both enhanced and weakened connectivity depending on the network or networks being examined, the context in which connectivity is being observed (i.e. at rest or in response to a task), and the specific characteristics of the study and sample in which these patterns emerged. At this stage of the research, it is difficult to determine whether these complex patterns are meaningful or represent artifacts that are unrelated to behaviors known to co-occur with or be related to obesity. Further, few studies have examined task-evoked and task-independent intrinsic functional connectivity within the same sample. This approach would provide some indication as to whether obesity-related abnormalities in functional connectivity are constrained to the evaluation of food or represent more fundamental, context-independent differences in the functional organization of the brain. Relatedly, a majority of studies to examine the relationship between obesity and network connectivity evoked by a specific stimulus or task have used food cues. Although this approach provides valuable information about how networks respond to food cues among individuals with overweight and obesity, it remains unclear whether the network disruptions summarized above extend to other potentially relevant cognitive domains that may be affected in obesity (e.g. executive control, emotion regulation and attention). Future studies should consider including an extensive cognitive, psychological and behavioral assessment battery to help contextualize neuroimaging results in obesity-relevant processes. Likewise, identifying modifiable factors that moderate the relationship between body mass and functional connectivity (e.g. sleep disruption, life stress, depressive symptoms and physical activity) will allow for better understanding of individual differences in vulnerability. More thorough characterization of research samples has the potential to facilitate the identification of subgroups within the clinical construct of obesity based on phenotypic differences in one or more domains, an important advancement that would not only generate new hypotheses about the etiology/etiologies of obesity but also help to refine and personalize treatments according to individual functional profiles.

An additional limitation is that there have been relatively few studies to examine the impact of dietary intake and eating patterns on functional network organization, leaving it unclear how the major behavioral determinant of obesity influences functional characteristics of the brain and vice versa. Although rigorous assessment of dietary intake remains challenging, it will be important to explore how both nutritional content of diet and patterns of eating affect functional networks in obesity so as to refine dietary interventions and guidelines. Finally, a crucial limitation of the existing literature is that the majority of research examining the relationship between obesity and functional network organization has been cross-sectional, aimed at first establishing whether and how obesity relates to network signaling dynamics. At present, the limited availability of intervention and longitudinal studies of functional connectivity as it relates to weight gain and weight loss precludes the drawing of any firm inferences about whether excess weight is the cause or consequence of changes in functional network dynamics. This in turn limits the translation of findings to the clinical context, impeding efforts to mitigate rising rates of obesity and obesity-related disease. In order to determine the direction of causality between obesity and functional network organization, it will be imperative to conduct longitudinal and intervention research to monitor the temporal dynamics of changes in weight and changes in the brain over time, including during childhood and adolescence prior to the development of obesity. It will also be imperative to include extended follow-up assessments of individuals who have participated in a weight loss intervention to determine whether weight loss-induced variation in functional network characteristics predicts individual differences in weight regain following the cessation of the active phase of intervention. Using a development framework to assess the relationship between functional brain networks and obesity across the lifespan is critical to enhancing understanding of the mechanisms underlying the development and maintenance of obesity, as well as weight regain following successful weight loss, information which may be leveraged to improve the efficacy of treatment and prevention.

## Conflicts of interest

None declared.

## Funding

This research was supported by National Institutes of Health (R01 DK095172 [PI: Kirk Erickson]; T32HL007560 [PI: Karen Matthews; awardee: Shannon Donofry]). The funding sources had no involvement in the writing of the manuscript or in the decision to submit the manuscript for publication.
